# Struck Oil

**Published:** 1864-12

**Authors:** 


"STRUCK OIL."—The Philadelphia correspondent of the *Boston Medical Journal*, says: Stop! We have struck oil. That is, as a profession. It is astonishing to what an extent the profession are going into oil stocks. They speculate in oil, think and drink oil, prescribe oil, and—what do you think of the following:—℞ 100 Walnut Island, *a* 5½ b. 30. M. It is a fact. At all the meetings, clubs, everywhere, it is oil! oil!! oil!!! One quite prominent M. D. is said to have made $15,000 in a few weeks. I am afraid, at this rate, the fees will be so lubricated that they will slip *out* of our hands faster than *in*, and the poor patients will slip through our fingers into the hands of the undertaker.

